# Age Is Only a Number Also in Hyperacute Stroke Care—But Not an Irrelevant One

**DOI:** 10.3390/jcm11164737

**Published:** 2022-08-13

**Authors:** Jussi O. T. Sipilä

**Affiliations:** 1 Department of Neurology, North Karelia Central Hospital, Siun Sote, 80210 Joensuu, Finland; jussi.sipila@utu.fi; 2 Clinical Neurosciences, University of Turku, 50520 Turku, Finland

“It is difficult to make predictions, especially about the future.” This we all know, but the phrase is particularly spot-on regarding the prognostication of individual patients. Yet, clinicians are required to make these predictions every day, often with insufficient background data or time to communicate with the patient—let alone contemplate the situation. Since time is brain, modern hyperacute stroke care is perhaps the best example.

The acute care of patients who have experienced ischemic stroke has advanced tremendously in the past two decades. Thrombolysis with tissue plasminogen activator (tPA) has become standard hyperacute therapy. More recently, endovascular thrombectomy (EVT) has become an even more powerful tool that is used in cases when there is an observable large vessel occlusion (LVO) and potentially salvageable brain tissue [1]. However, this therapy is only available in comprehensive stroke centers and requires advanced neurovascular expertise in its provision and patient selection. It is also uncertain whether EVT is better used with or without preceding thrombolysis, and the efficacy of both of these therapies is highly time-dependent [2,3,4,5,6]. Therefore, these advancements have created new challenges in the design of efficient provision strategies regarding acute stroke care services.

Some uncertainty also exists regarding patient selection, as large, controlled trials excluded many (or included very few) patients with situations often encountered in clinical practice. One of these uncertainties concerns patient age. While it is now clear that the elderly can also benefit from both tPA and EVT, they seem to gain less from these treatments than younger patients [7,8,9,10,11,12]. Additionally, although at least in Finland the elderly are in better shape than has previously been the case, it is also clear that, in general, older patients have a poorer prognosis and, in particular, shorter post-stroke survival times compared to younger patients [13,14,15,16]. Therefore, it is even more important to evaluate individual prognoses in elderly patients.

How can this be achieved? While we wait for randomized trials to clarify the situation [17], some clues exist. The SPAN-100 index is perhaps the most obvious one: if patient age + NIHSS score ≥ 100, the outcome is very likely poor [18,19,20]. On the other hand, as with younger people, some elderly individuals are in better shape than others, so biological age should be considered instead of chronological. One potential biological indicator is frailty, which is a common and important survival predictor in the elderly with or without a stroke [21,22]. Therefore, routine frailty evaluation in acute stroke patients seems to be needed to inform treatment decisions. However, there are many ways to assess frailty, and the suitability of these methods to the hyperacute stroke setting is unclear. Ideally, frail people would be routinely identified by geriatricians and GPs, and this information would be ready and available in the hyperacute setting. As work remains to be done on this, the development and clinical validation of a digital tool utilizing patient record data from previous healthcare encounters could be an intermediate solution [23]. Compared to large, randomized studies evaluating hyperacute interventions, it seems that the investigation and implementation of these assessments can be more easily and swiftly conducted. Therefore, it is likely that targeted interventions to improve knowledge on the signs and risk factors of strokes, specifically in elderly populations with lower educational levels, could also be easily and quickly implemented, which might lead to faster admissions and even lower stroke incidences [24]. Interventions are also needed among the younger population to reduce the expected stroke burden in the future elderly population [25,26]. Ultrasound techniques for the pre-hospital identification of LVO in octogenarians should also quickly be evaluated because, in a drip-n-ship setting, these patients might particularly benefit from being transferred directly to an EVT-capable center [6,27]. The strategy of skipping tPA in EVT-eligible LVO patients with a high risk of hemorrhage may also be considered [3].

Acute hospitalization often leads to deterioration in the elderly [28,29]. Fortunately, patients of all ages benefit from care in a stroke unit [30,31], where the first steps can be taken in order to try to achieve their prior functional status. However, no neurological procedure can help a patient achieve a better functional status compared to the one preceding the stroke, and therefore, information on this should be easily available to aid decision making. This highlights the importance of co-operation not only between specialized care providers but also with primary healthcare. User-friendly, comprehensive electronic medical record systems are also needed.

Higher age, prior functional dependence and comorbidities are all related to patients’ abilities to benefit from revascularization treatment and need to be taken into account in decision making. Endovascular treatment options may also continue to improve outcomes in the elderly, but more data are needed regarding the optimal treatment pathways [6]. Optimal results can only be achieved with close collaboration using modern equipment and up-to-date scientific and individual patient data.
